# Recommendations for cardiovascular magnetic resonance and computed tomography in congenital heart disease: a consensus paper from the CMR/CCT working group of the Italian Society of Pediatric Cardiology (SICP) and the Italian College of Cardiac Radiology endorsed by the Italian Society of Medical and Interventional Radiology (SIRM) Part I

**DOI:** 10.1007/s11547-022-01490-9

**Published:** 2022-05-24

**Authors:** Aurelio Secinaro, Lamia Ait-Ali, Davide Curione, Alberto Clemente, Alberto Gaeta, Andrea Giovagnoni, Annalisa Alaimo, Antonio Esposito, Bertrand Tchana, Camilla Sandrini, Elena Bennati, Emanuela Angeli, Francesco Bianco, Francesca Ferroni, Francesca Pluchinotta, Francesca Rizzo, Francesco Secchi, Gaia Spaziani, Gianluca Trocchio, Giuseppe Peritore, Giovanni Puppini, Maria Cristina Inserra, Nicola Galea, Nicola Stagnaro, Paolo Ciliberti, Placido Romeo, Riccardo Faletti, Simona Marcora, Valentina Bucciarelli, Luigi Lovato, Pierluigi Festa

**Affiliations:** 1 Advanced Cardiothoracic Imaging Unit, Department of Imaging, Bambino Gesù Children’s Hospital, IRCCS, Rome, Italy; 2 Pediatric Cardiology and GUCH Unit, Fondazione “G. Monasterio” CNR-Regione Toscana, Massa-Pisa, Italy; 3 Department of Radiology, Fondazione “G. Monasterio” CNR-Regione Toscana, Massa-Pisa, Italy; 4 Pediatric Radiology Unit, Giovanni XXIII Children’s Hospital, Bari, Italy; 5 Radiology Department, Azienda Ospedaliero-Universitaria Ospedali Riuniti Ancona “Umberto I, G. M. Lancisi, G. Salesi”, Ancona, Italy; 6 U.O.C. Cardiologia Pediatrica, P.O. “G. Di Cristina”, ARNAS Civico, Palermo, Italy; 7 Clinical and Experimental Radiology Unit, Experimental Imaging Center, IRCCS Ospedale San Raffaele, Milan, Italy; 8 Pediatric Cardiology Unit, General and University Hospital, Parma, Italy; 9 Division of Cardiology, Department of Medicine, University of Verona, Verona, Italy; 10 Pediatric Cardiology, Azienda Ospedaliero-Universitaria Meyer, Florence, Italy; 11 Pediatric and Grown-Up Congenital Cardiac Surgery Unit, IRCCS Azienda Ospedaliero-Universitaria di Bologna, Bologna, Italy; 12 Department of Paediatric and Congenital Cardiac Surgery and Cardiology, Azienda Ospedaliero-Universitaria Ospedali Riuniti Ancona “Umberto I, G. M. Lancisi, G. Salesi”, Ancona, Italy; 13 Division of Pediatric Cardiology, Città della Salute e della Scienza, Turin, Italy; 14 Department of Pediatric Cardiology and Adult Congenital Heart Disease, IRCCS Policlinico San Donato, Milan, Italy; 15 Radiology Unit, Giannina Gaslini Research Institute and Children Hospital, Genoa, Italy; 16 Department of Radiology, IRCCS Policlinico San Donato, Milan, Italy; 17 Pediatric Cardiology Department, Giannina Gaslini Research Institute and Children Hospital, Genova, Italy; 18 U.O.C. di Radiodiagnostica, P.O. “G. Di Cristina”, ARNAS Civico, Palermo, Italy; 19 Department of Radiology, University of Verona, Verona, Italy; 20 Department of Radiology, University Hospital Vittorio Emanuele Catania, Catania, Italy; 21 Department of Experimental Medicine, “Sapienza” University of Rome, Rome, Italy; 22 Department of Cardiology and Cardiosurgery, Bambino Gesù Children’s Hospital, IRCCS, Rome, Italy; 23 Department of Surgical Sciences, Radiology Unit, University of Turin, Turin, Italy; 24 Department of Pediatric Cardiology, Papa Giovanni XXIII Hospital, Bergamo, Italy; 25 Pediatric and Adult Cardiovascular, Thoraco-abdominal and Emergency Radiology Unit, IRCCS Azienda Ospedaliero-Universitaria di Bologna, Via Massarenti 9, 40138 Bologna, BO Italy; 26 Department of Biomedical Sciences for Health, Università degli Studi di Milano, Milan, Italy

**Keywords:** Cardiovascular magnetic resonance, Cardiovascular computed tomography, Congenital heart disease, Pediatric cardiology, Multimodality imaging

## Abstract

Cardiovascular magnetic resonance (CMR) and computed tomography (CCT) are advanced imaging modalities that recently revolutionized the conventional diagnostic approach to congenital heart diseases (CHD), supporting echocardiography and often replacing cardiac catheterization. Nevertheless, correct execution and interpretation require in-depth knowledge of all technical and clinical aspects of CHD, a careful assessment of risks and benefits before each exam, proper imaging protocols to maximize diagnostic information, minimizing harm. This position paper, written by experts from the Working Group of the Italian Society of Pediatric Cardiology and from the Italian College of Cardiac Radiology of the Italian Society of Medical and Interventional Radiology, is intended as a practical guide for applying CCT and CMR in children and adults with CHD, wishing to support Radiologists, Pediatricians, Cardiologists and Cardiac Surgeons in the multimodality diagnostic approach to these patients. The first part provides a review of the most relevant literature in the field, describes each modality's advantage and drawback, making considerations on the main applications, image quality, and safety issues. The second part focuses on clinical indications and appropriateness criteria for CMR and CCT, considering the level of CHD complexity, the clinical and logistic setting and the operator expertise.

## Introduction

The constant improvements in surgical and interventional techniques have drastically increased the survival rate of congenital heart diseases (CHD) patients over the last decades, with an 85% estimate of children with CHD surviving to adulthood [1].

Imaging techniques are crucial in the multidisciplinary approach to these patients, characterizing anatomical structures and their functional status in order to improve management and guide pre and post-operative evaluation, lifelong surveillance and prognosis. Complementary use of various imaging modalities aims at incrementing accuracy, reproducibility, and cost-effectiveness while minimizing risks [1, 2].

Echocardiography is the first diagnostic tool in the preoperative evaluation and follow-up of CHD [3, 4]. However, it may be limited due to poor acoustic windows for the characterization of extracardiac anatomy and complex cases.

In the last decades, cardiovascular magnetic resonance (CMR) and computed tomography (CCT) have revolutionized the diagnostic approach to CHD [5], being an useful adjunct to echocardiography in multiple instances and they have replaced cardiac catheterization for many diagnostic indications [6–8]. Hence, good knowledge of their potential, limitations, and indications is mandatory [9–11]. Yet, some indications are still a matter of discussion.

The present consensus, proposed by the CMR/CCT working group of the Italian Society of Pediatric Cardiology and by the Italian College of Cardiac Radiology of the Italian Society of Medical and Interventional Radiology (SIRM), is addressed to Radiologists, Pediatricians, Cardiologists and Cardiac surgeons, interested in CHD imaging. It is divided into two parts. The first one provides a review of the most relevant literature in the field, a description of each modality’s advantages and drawbacks, and a comment on future technological perspectives. The aims are:

1. To provide an overview of the technical advantages and disadvantages of CMR and CCT in CHD;
2. To propose clinical recommendations, based on patient's age, CHD complexity, and the required operator expertise.

Further clinical issues are treated in the second part with a specific description of CCT and CMR appropriateness criteria based on this proposed novel approach.

## Review of the literature on the indications of CMR and CCT in CHD

From the early ‘80 s several reports and guidelines have been published in this field, progressively attributing a more fundamental role to CMR and CCT in CHD [1, 6, 7].

In 2015, an European Society of Cardiology (ESC) consensus paper was published [9], based on expert opinions concerning CMR use in children with CHD in specific clinical situations, while the same year two expert consensus documents of the Society of CCT were published [10, 11]. Part 1 offered a systematic review of all indications and risks related to CCT in CHD. Part 2 described the optimal technical environment and protocols and the most relevant knowledge for CCT performance in CHD.

In 2018, the European Association of Cardiovascular Imaging released a position paper focused on the multimodality imaging approach to adult CHD [5]. This leading role of CMR and CCT is also established by the American College of Cardiology/American Heart Association (ACC/AHA) guidelines [12, 13]. The latest update re-emphasized how cardiac imaging must be supervised and interpreted by physicians with expertise and/or training in CHD [13]. Additionally, in 2020 the ACC/AHA published appropriateness criteria for using multimodality imaging during the follow-up care of CHD patients [14]. More recently, the new European guidelines for the management of adult CHD by the ESC were published [1], followed by two expert consensus documents by Radiologists and Cardiologists about the appropriateness criteria of CCT/CMR use in different clinical scenarios including CHD, endorsed by the SIRM and by the Italian Society of Cardiology [8, 15].

## Strengths and limitations of CMR and CCT in CHD

CMR and CCT imaging have significantly changed the diagnostic approach to CHD, limiting the need for invasive procedures.

**Fig. 1:**
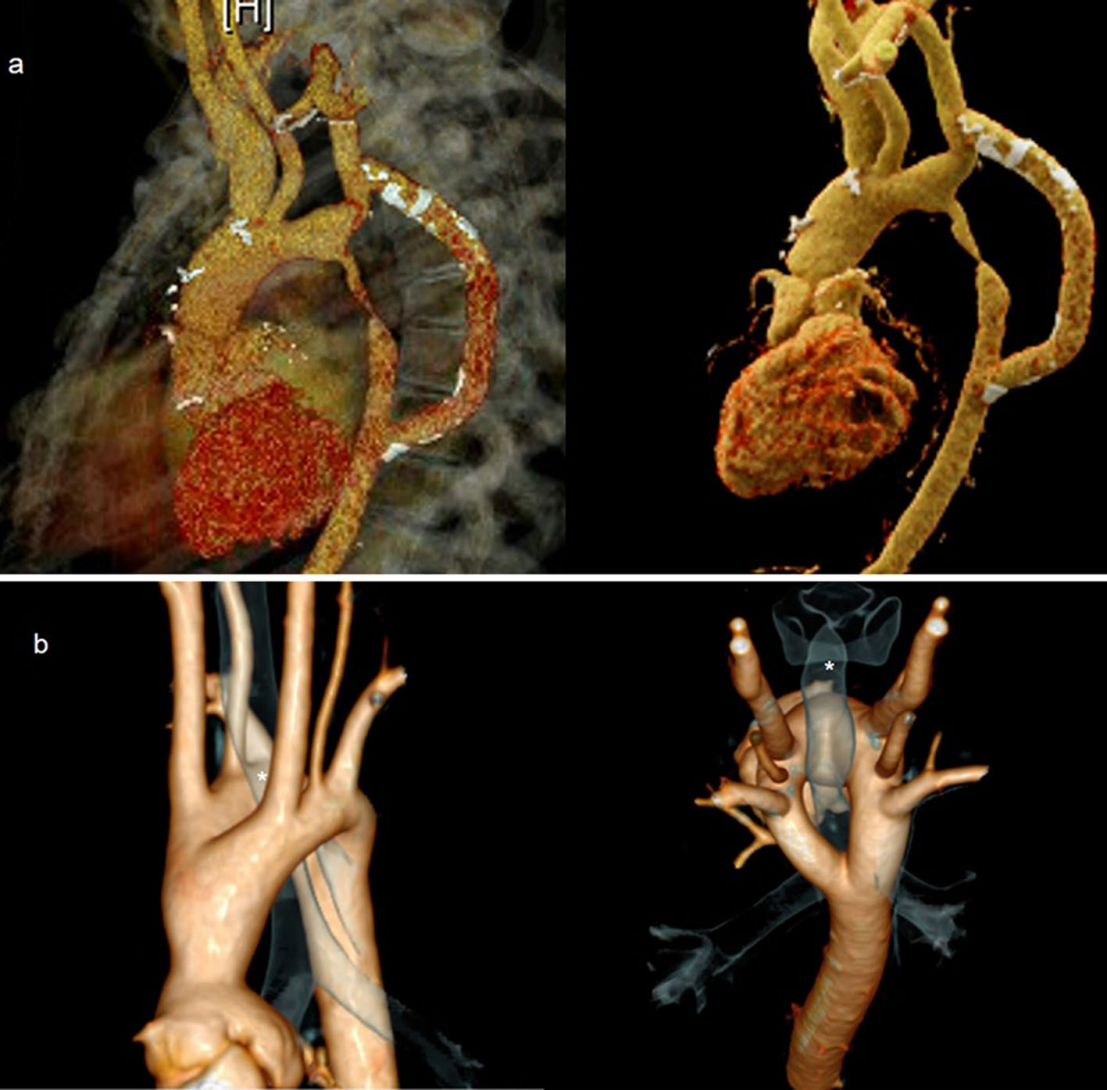
CCT 3D volume rendering images, sagittal view of a 54-year-old woman with bypass aortic coarctation palliation (**a**). CCT 3D volume rendering images of a double aortic arch with 3D rendering of airways structures (asterisks) showing anatomical relationships between them and the vascular ring (**b**). *CCT* Cardiovascular computed tomography, *3D* tridimensional

**Fig. 2:**
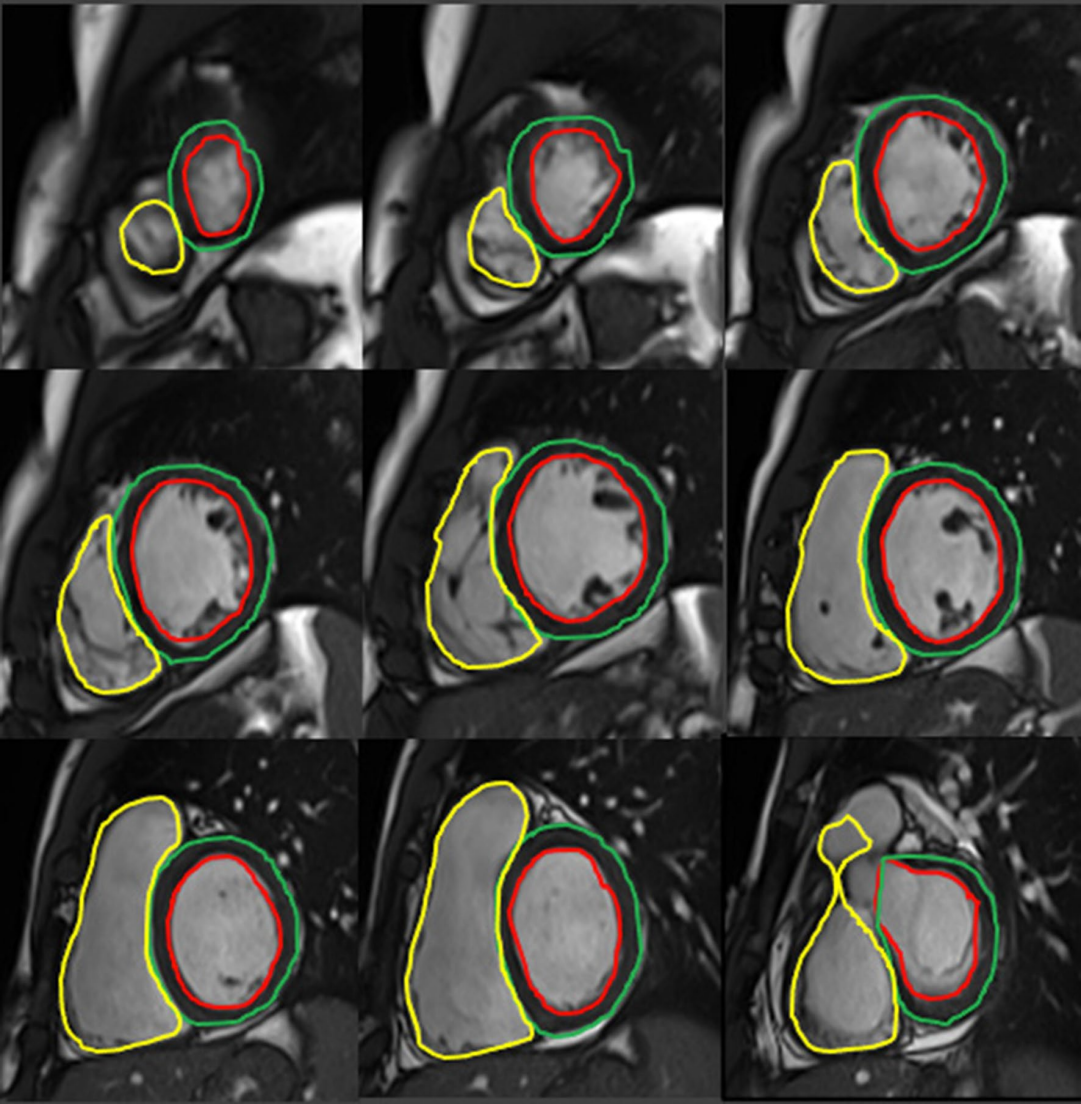
CMR quantification of biventricular volumes through semiautomatic delineation of epicardial and endocardial borders in a ventricle base to apex stack of short axis slides: the absence of geometric assumptions and panoramicity assures optimal reproducibility and high measurement accuracy

Some advantages are common to both techniques. Unlike echocardiography, they are not restricted by body habitus, acoustic windows or geometric assumptions. One of their most notable features is the superb three-dimensional (3D) capability, which allows for excellent visualization of cardiovascular structures with high spatial and temporal resolution (Fig. 1). This is especially useful when planning percutaneous or surgical interventions [5]. Moreover, CMR and CCT have proven very low intra/inter-observer variability in CHD assessment, with particular regard to biventricular function, volumes and mass quantification [16–18] (Fig. 2).

On the other hand, limited availability, high costs, and the need for specific hardware/software equipment and expertise are the main drawbacks to their widespread use in CHD [13, 19, 20]. The risks of CMR and CCT are summarized in Table 1, while a summary of the main advantages and drawbacks of each imaging modality is described in Table 2.

**Table 1:** Risk and hazard comparison between CMR and CCT

Risks and hazards	CMR	CCT
Biological effects	Unknown cancer risk of non-ionizing EMF	Cancer risk of ionizing radiation
Genotoxic effects	Damage should be reversible	–
RF field	Heating/Burns	–
Gradient field	Loud noise/peripheral nerve stimulation/induced voltages in abandoned PM wires or long conducting implants	–
Main magnetic field	Magnetic force and torque on:	–
Fm external devices	Ferromagnetic projectiles (tools, beds, stretchers…)	–
Fm implanted devices	Electromagnetic field interactions with devices and artifacts	–
Sedation or general anesthesia	Limits intrinsic thermoregulation	–
Contrast medium	Not always necessary	Necessary
	NSF	Allergic-like reactions
	CI renal failure	CI renal failure

*CCT* cardiovascular computed tomography, *CI* contrast induced, *CMR* cardiovascular magnetic resonance, *EMF* electro-magnetic fields, *Fm*, ferromagnetic, *NSF* nephrogenic systemic fibrosis, *PM* pacemaker, *RF* radiofrequency

**Table 2:** Advantages and disadvantages of CMR and CCT in CHD

	CMR	CCT
Advantages	Combined functional and morphological information	Fast acquisition
	Accurate ventricular volumes and vessel flow quantification	Good temporal resolution
	No ionizing radiation	Limited artifacts due to movement and fast heart-rates
	High Temporal resolution (up to 30 ms)	Highly detailed information on vascular anatomy
	Non-contrast scan	Accurate delineation of coronary anomalies
	3D images feasible and accurate	Functional data (retrospective ECG-gated scan)
	Accurate information on vascular anatomy	
	Detection of coronary anomalies	
Disadvantages	Longer scan time (from 40 min on)	Radiation exposure
	Suboptimal imaging in case of arrhythmias	Suboptimal imaging in case of arrhythmias
	More susceptible to respiratory artifacts	Iodinated contrast always needed
	Need for general anesthesia for non-cooperative pts	
	Limited access for metallic implants or claustrophobia	
	Gadolinium adverse events (NSF, Brain Deposits)	
Temporal resolution	~ 30 ms	66–75 ms (DSCT)
		140–150 ms (single source CT)
Spatial resolution	~ 0.9–1 mm (voxel size)	~ 0.4 × 0.4 × 0.6 mm

*CCT* cardiovascular computed tomography, *CMR* cardiovascular magnetic resonance, *DSCT* dual source computed tomography, *NSF* nephrogenic systemic fibrosis

## Cardiovascular magnetic resonance

**Fig. 3:**
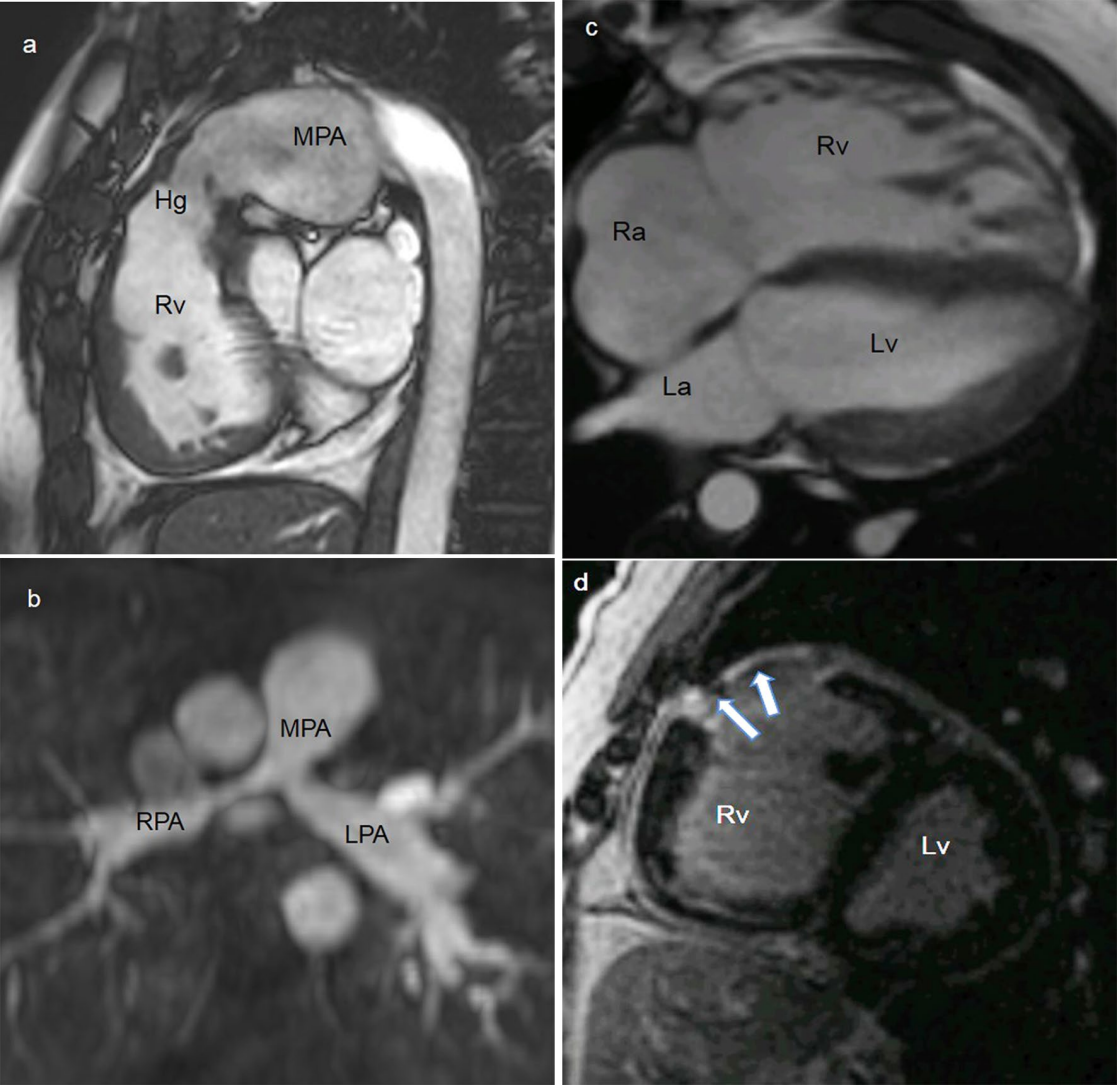
30-year-old female with repaired Tetralogy of Fallot (ToF). CMR SSFP image, RVOT sagittal plane shows a slightly reduced homograft caliber with post-stenosis pulmonary artery dilation (**a**). 21-year-old male after aortic coarctation and VSD repair. MRA MPR axial image shows residual pulmonary bifurcation and proximal branch arteries stenosis post pulmonary banding (**b**). 31-year-old female with repaired ToF. CMR SSFP 4-chamber view demonstrates right ventricular dilatation (**c**). CMR LGE short-axis view shows RVOT post-surgical scar (arrows) (**d**). *CMR* cardiovascular magnetic resonance, *Hg* Homograft, *La* left atrium, *LPA* left pulmonary artery, *LGE* late gadolinium enhancement, *Lv* left ventricle, *MPA* pulmonary artery, *MPR* Multiplanar Reformation, *MRA* Magnetic Resonance Angiography, *Ra* right atrium, *RPA* right pulmonary artery, *Rv* right ventricle, *RVOT* right ventricle outflow tract, *SSFP* Steady State Free Precession, *VSD* Ventricular septal defect

CMR is the only imaging modality offering in a single step exam excellent anatomical pictures of cardiovascular structures and functional/hemodynamic information [5, 7]. CMR is justified in each patient with insufficient clinical or echocardiographic data for monitoring, decision-making, or treatment planning [1], and in complex CHD to guide clinical decisions [9]. Lifelong follow-up with serial CMR imaging is also recommended in grown-up congenital heart disease (GUCH) patients [1], often submitted to surgical repairs during childhood (Fig. 3), for its high reproducibility and limited safety issues compared to CCT and/or catheterization, considering the relatively young population.

To date, CMR is considered the gold standard for volumes and myocardial mass assessment, especially of the right ventricle (RV) (i.e., cine imaging) and highly appropriate for flow and shunt quantification, allowing for hemodynamic assessment of valvular pathology (i.e., phase-contrast sequences). Nevertheless, CMR is not superior to echocardiography in estimating gradients or evaluating atrio-ventricular valvular and sub-valvular pathology and remains inferior in detecting small mobile structures like vegetations or patent foramen ovale [1, 21]. Moreover, CMR can precisely delineate intra and extra-cardiac anatomy by means of several sequences (i.e., black-blood spin echo, Magnetic Resonance Angiography, 3D steady-state free-precession). A further strength of CMR is its ability to perform tissue characterization, achieved with late gadolinium enhancement (LGE) sequences [5]. The acquisition of LGE-CMR, even though not always required in CHD and not to be repeated at each imaging follow-up examination, is a considerable integration to morpho-functional evaluation, allowing for the identification of focal myocardial fibrosis that has been associated to adverse cardiac events (i.e., heart failure, cardiac arrhythmias) in many CHD.

CMR can be performed on either 1.5 or 3 Tesla (T) scanners, with 1.5 T being the clinical standard due to more robust scanning sequences [22].

CMR has broadly a high safety profile, even in children. The lack of radiation exposure and options to avoid contrast administration in many cases are well-established advantages, characterizing its favorable benefit/risk profile compared with catheterization and CCT [23, 24]. Nonetheless, CMR involves exposure to electromagnetic energy (static magnetic fields, gradients and radiofrequency pulses), with intrinsic hazards from ferromagnetic external (i.e., "projectile effect") and/or implanted devices, induced electrical currents, heating, and acoustic noise. Therefore, a safety screening should be performed prior to every exam to rule out contraindications, including non-MR-compatible pacemakers (PMs), implantable cardioverter defibrillators (ICDs), cochlear implants, and other ferromagnetic devices [19, 22]. Most of implanted devices do not represent an absolute contraindication to CMR being classified as CMR-conditional. Nevertheless, CMR-conditional cardiac devices require collaboration with the electrophysiology team for CMR execution and may produce significant artifacts [25, 26]. Remarkably, safe protocols have recently been shown feasible with conventional PMs and ICDs [27, 28].

Despite recent technical advances, CMR is still time-consuming, especially in complex cases [29, 30]. Consequently, young children and uncooperative patients require deep sedation with spontaneous respiration or general anesthesia [1, 5], needing CMR-compatible equipment. CMR has proven to be safe even during general anesthesia in fragile subjects, when adopting a consolidated approach by an experienced multidisciplinary team [31, 32]. Patients affected by complex CHD or Williams syndrome deserve special consideration, because of an increased risk of adverse events during sedation [33, 34].

Intravenous administration of gadolinium-based contrast agents (GBCAs) is commonly performed in CHD, although not mandatory except for tissue characterization, myocardial perfusion or contrast-enhanced angiography. GBCAs can be considered safe in both children and adults [35], as adverse events are very rare and usually mild. A relative contraindication exists for severe kidney disease (glomerular filtration rate < 30 mL/min/1.73m^2^) due to the risk of nephrogenic systemic fibrosis, a rare but serious condition that can compromise internal organs [22]. Recently, the potential long-term effects of gadolinium deposition in tissues, particularly in the brain, have aroused considerable interest although its real clinical relevance still needs more research [36]. For the abovementioned reasons, since 2018 the European Medicines Agency and subsequently the corresponding Italian authority suspended the distribution of linear non-ionic GBCAs and limited the use of linear ionic agents. Therefore, macrocyclic ionic molecules are those allowed for cardiovascular MR scans.

## Cardiovascular computed tomography

CCT has been increasingly used as a non-invasive imaging modality in CHD patients of all ages (15). Current generation multi-slice CCT scanners allow for rapid coverage of large anatomic volumes with excellent spatial and temporal resolution, overall short examination times, and low radiation exposure [10].

Wider availability and lower cost compared to CMR have also favored its success.

**Fig. 4:**
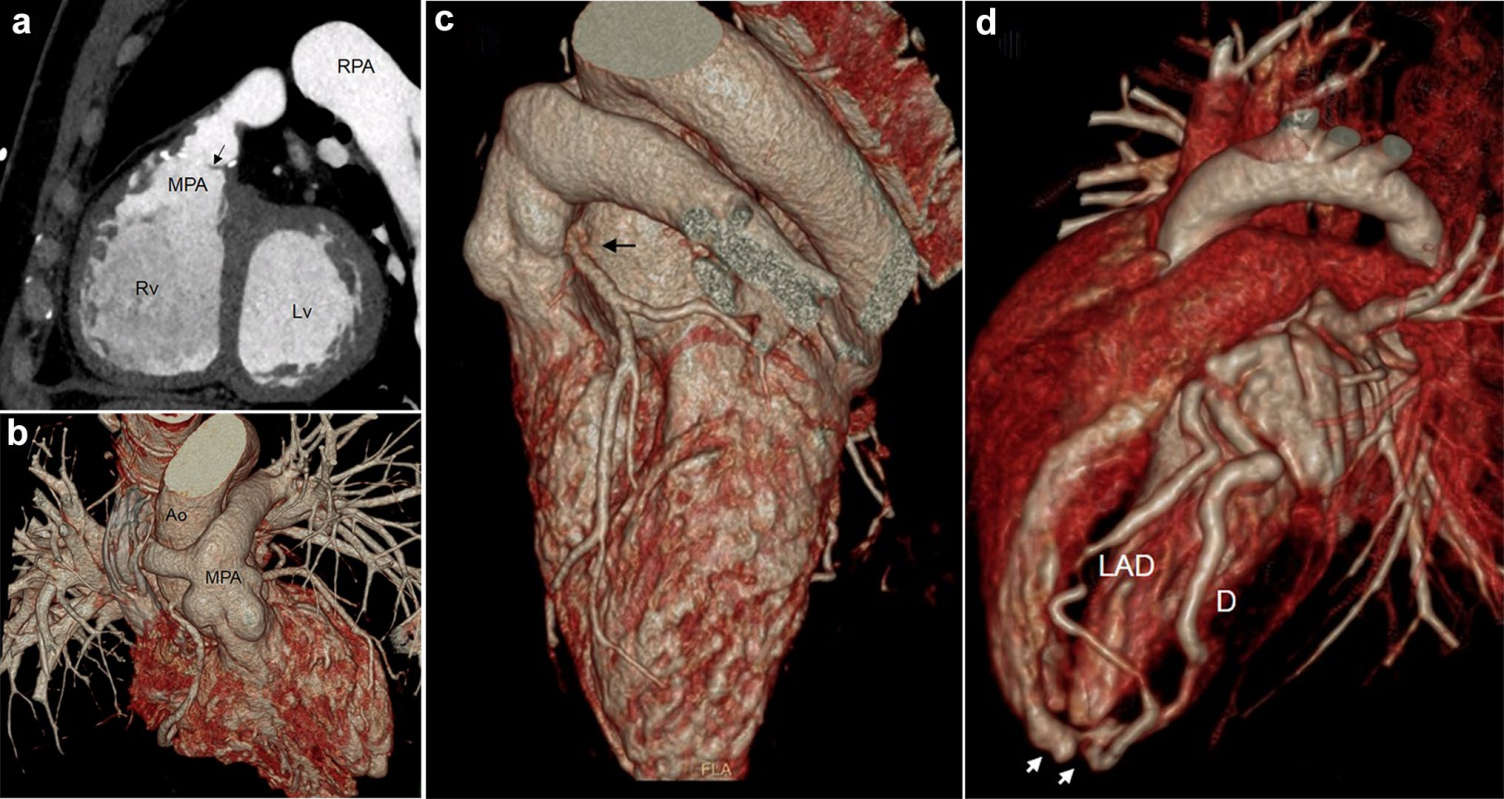
31-year-old male with a repaired ToF. CCT MPR image, RVOT sagittal plane: infundibular and pulmonary stenosis (**a**), note the anatomical detail of valvular cusp (arrow). 16-year-old female with a cTGA after Arterial Switch operation. CCT 3D volume rendering images demonstrate high resolution post-surgical anatomy (**b**), LCA reimplantation kinking and stretching (black arrow) is well depicted (**c**). 2-year-old child with coronary artery fistula. CCT 3D volume rendering image optimally shows the fistula (white arrows) between LAD artery and Rv chamber (**d**). *Ao* Ascending aorta, *CCT* Cardiovascular computed tomography, *cTGA* Complete transposition of the great arteries, *3D* Tridimensional, *D* Diagonal artery, *LAD* Left Anterior descending artery, *LCA* Left coronary artery, *Lv* Left ventricle, *MPA* Main pulmonary artery, *MPR* Multiplanar Reformation, *RPA* Right pulmonary artery, *Rv* Right ventricle, *ToF* Tetralogy of Fallot

CCT is an alternative to other imaging tools, when they cannot provide good quality images, when CMR is contraindicated or present logistic difficulties such as anesthesia [11, 37]. CCT, due to its higher sub-millimetric isotropic spatial resolution (up to 0.4 × 0.4.0.6 mm for modern multirow detectors scanners), is considered superior to CMR in cardiovascular anatomy delineation (Fig. 4), primarily for small blood vessels analysis such as coronary arteries, collaterals, fistulas, and arteriovenous malformations [20]. Electrocardiographic (ECG)-synchronization is mandatory only for coronary, aortic root, and/or intracardiac imaging. Besides, CCT enables optimal depiction of airways and lung parenchyma and it is the preferred imaging modality when tracheobronchial or pulmonary abnormalities need to be investigated [5, 10, 11].

Like CMR, CCT performance is strongly dependent on scanner technology: detector number and size, gantry rotation speed, double source technology, temporal resolution (66–75 ms for dual source scanners and 140–150 ms for single source machines) and dose reduction algorithms significantly influence its accuracy and applicability, especially in newborns/infants and coronary artery imaging. Another major advantage of CCT over CMR is its shorter duration, and no need for specific equipment for sedation, particularly useful in uncooperative patients, namely younger children and critically ill subjects in the acute setting [38]. High pitch (up to 3.4 for dual source scanners) or target mode acquisition with newer generation scanners permits image acquisition in a single or few heartbeats, acquiring data during a small cardiac cycle portion (i.e., prospective scanning) [39, 40], thus drastically diminishing cardiac and respiratory motion artifacts and provide diagnostic images even at higher heart rates encountered in neonates and infants [11]. Consequently, exams performed for most indications can be obtained without sedation and even during free breathing [41, 42] while the use of beta-blockers or nitrates is reduced or eliminated unless detailed coronary artery imaging is sought. In contrast, older generation scanners may be inadequate for some indications, with an increased need for sedation and pre-medication [11].

**Fig. 5:**
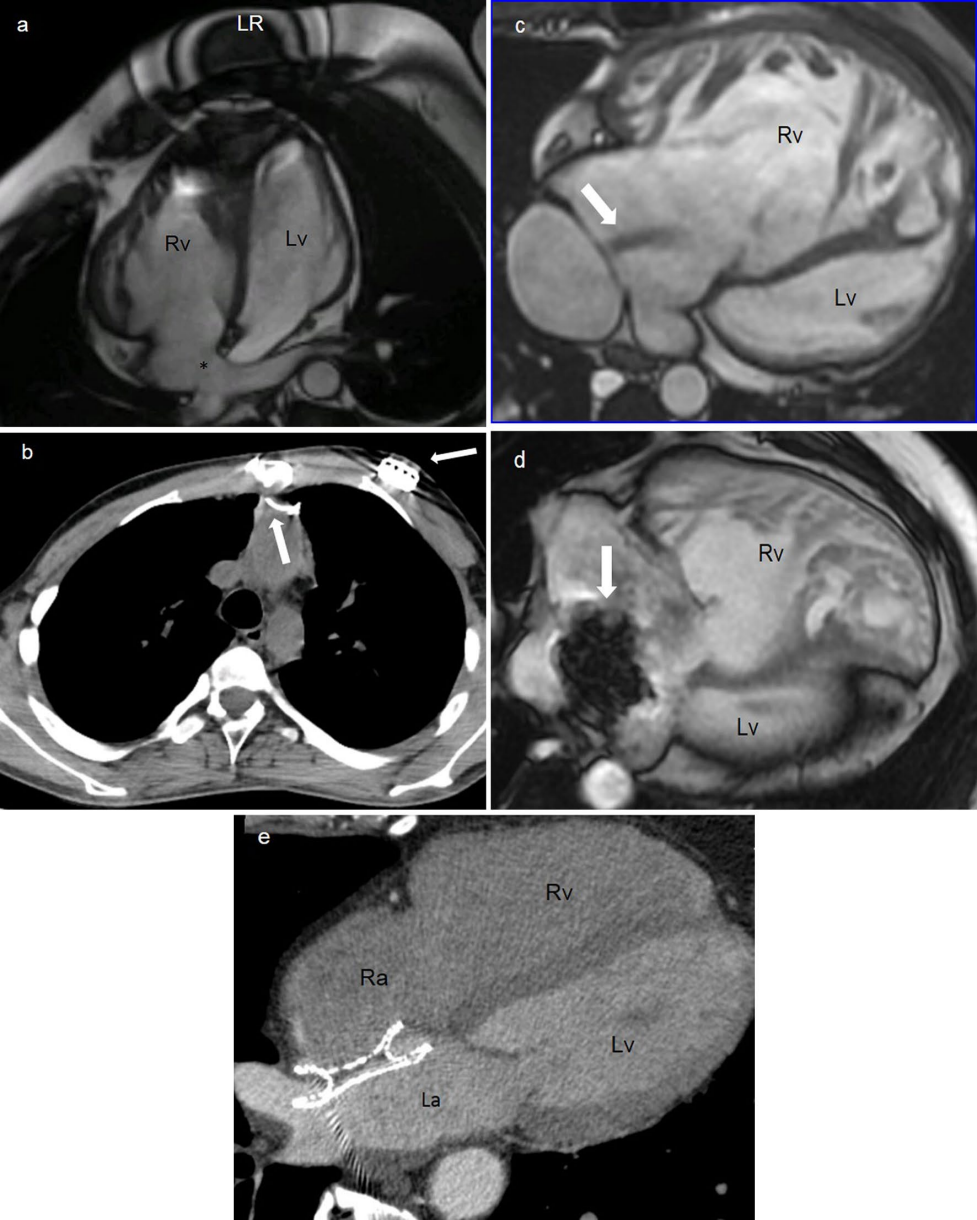
34-year-old male with Mustard repair of c-TGA and loop recorder implantation. CMR SSFP 4-chamber view shows atrio-ventricular concordance and the pulmonary baffle (asterisk). The ventricular apex is canceled by artifacts (**a**). 18-year-old Fontan patient with pacemaker implantation. CCT axial plane displays the wires and the pacemaker generator (arrows) with minimal artifacts upon thoracic aorta (**b**). Adult male patient with Mustard repair of c-TGA and baffle leakage. CMR SSFP 4-chamber images show the flow turbulence (arrow) before the treatment (**c**) and a huge artifact (arrow) caused by the metallic closure device (**d**). Adult male with ASD after endovascular closure. CCT axial image well depicts the closure device without limitations to cardiac chambers visualization (**e**). *CCT* cardiovascular computed tomography, *CMR* cardiovascular magnetic resonance, *cTGA* complete transposition of the great arteries, *SSFP* Steady State Free Precession, *La* left atrium, *Lv* left ventricle, *Ra* right atrium, *Rv* right ventricle

CCT is less susceptible than CMR to metallic artifacts (Fig. 5). Although stents are not a contraindication to CMR, CCT is superior in diagnosing stent patency and integrity [43] and it is preferable when evaluating metallic devices and calcifications within conduits and vessels [3, 5]. Furthermore, CCT is not limited by implanted cardiac devices [1, 5].

CCT accurately quantifies right and left ventricular volumes and function [44, 45], although with lower temporal resolution than CMR or echocardiography and at the expense of increased radiation exposure, needing ECG-synchronized data acquisition during the whole cardiac cycle (i.e., retrospective scanning). Therefore, it should be considered with caution for serial measurements, mainly when CMR is contraindicated or degraded by artifacts. Moreover, CCT does not provide hemodynamic information [20].

Obviously, the main drawback of CCT is radiation exposure, which is considered to increase the risk of future cancer development [46]. This is especially concerning in younger patients characterized by longer expected lifespan and greater radiation sensitivity. Recent advancements in scanner technology and reconstruction methods [47] have led to low-dose CCT protocols, which are now consistently inferior to cardiac catheterization with careful selection of scan parameters [48] and can currently be employed for many CHD indications (for many of which it can reach effective dose level equal to or lower than 1 mSv), except for coronary imaging where the exposure reduction is limited by several patients and scanner features.

CCT requires intravenous administration of iodine-based contrast agents. Non-ionic low or iso-osmolar agents are used because adverse reactions are rarer and usually milder, including allergic-type and non-allergic reactions similar to GBCAs [49]. Severe kidney disease is a contraindication to injection due to the risk of contrast-induced nephropathy [50].

## Comparative cost

The cost analysis of imaging modalities in CHD, specifically in children, rarely reaches adequate cost-effectiveness [51]. Overall costs often significantly exceed local reimbursements, particularly for complex examinations such as CMR under general anesthesia [52]. CMR scan costs in Western societies exceed several times those of echocardiography due to purchasing, running, and maintaining the scanner and staff expertise and training requirements. The decision to request a CMR and CCT investigation must be guided by the additional information needed, local facilities, and available resources for scanning [53]. Workflow and technical optimization (scan time, processing, and reporting) may be beneficial. In this sense, the combined use of rapid imaging, artificial intelligence algorithms [54], structured reports, and strategies to reduce the need for general anesthesia ("feed and wrap", MRI-safe audio–video systems for entertainment) could speed up the diagnostic process. On the other hand, CCT is faster and less expensive than MRI, often does not require general anesthesia, and can sometimes be preferred for its feasibility and availability, especially in clinical emergencies.

## Levels of recommendation of CMR and CCT in CHD

Based on the literature and expert opinions, this consensus paper proposes a novel approach to recommendations for CMR and CCT in CHD, divided into 3 levels, according to patient's age, disease complexity, and the required imaging experience. This model appears to be more suitable in routine clinical activity due to the extreme variability of the clinical scenarios and issues addressed in CHD [5, 10, 11, 13, 19]. The levels of recommendation are summarized in Table 3.

**Table 3:** levels of recommendation of CMR/CCT in CHD

	Level 1	Level 2	Level 3
Definition	*Cooperative Adolescent/adult patients with simple/moderate CHD that require additional cross-sectional imaging investigation*	*Cooperative patients with moderate/complex CHD candidate to longitudinal cross-sectional imaging evaluation*	*Uncooperative patients and complex CHD proposed for “optional” and technically difficult cross-sectional imaging investigation in potentially unsafe scanning condition*
Refer to	*Any center with cardiovascular imaging experience*	*Specialized center with experience in the diagnosis and treatment of CHD (Hub and Spoke)*	*Tertiary center with long-standing experience in the diagnosis and treatment of CHD (Hub)*
General indication	Non-complex CHD in cooperative patients	Follow-up of CHD in adolescents and adults	Complex CHD pre/post-repair (e.g., univentricular heart Fontan, atrial switch, isomerism)
	Native or repaired Aortic Coarctation	Non-complex CHD in children	Fragile patients
	Anatomy of PVR, anatomy of Ao Arch	Conotruncal anomalies post-repair (TOF, TGA post arterial switch, Truncus…)	Anesthesiologic difficulties (Williams syndrome patients requiring anesthesia)
	Pulmonary vascular disorders anatomy of ASD, VSD	Post Ross intervention	Technical difficulties (highly specific sequences/protocols/facilities)
	*CMR*	Ebstein/tricuspid dysplasia	
	Simple shunt quantification		
	Semilunar valve regurgitation		
	*CT*		
	Airway/lungs anomalies		
	Coronary anomalies*		

*Ao* aortic ASD, atrial septal defect, *AV* artero-venous, *CHD* congenital heart disease, *PVR* pulmonary venous return, *VSD* ventricular septal defect, *TOF* Tetralogy of Fallot, *TGA* transposition of the great arteries. The choice of CMR versus CCT depends on the information required for patient's management and local availability. When both can provide the same information with no added risks (i.e., anesthesia), CMR is preferable

^*^At least 64-rows or superior CT technology is required. Specific low-radiation dose CT equipment is highly preferable

**Level 1** *Adolescent/adult patients affected by simple to moderate complexity CHD that require additional cross-sectional imaging investigation with CMR and/or CCT → it refers to any center with cardiovascular imaging experience.*

This level includes native or repaired CHD of simple to moderate complexity in older children/adolescents and adults (e.g., aortic coarctation, pulmonary venous return or aortic arch anatomy, shunt quantification). The choice of imaging modality and timing of execution are well defined and widely acknowledged. Since patients are stable, cooperative, and do not necessarily require the latest generation technology, which is usually only available in highly specialized centers, they could be addressed to and/or managed by any center with CMR or CCT equipment and experience [10, 11, 55].

**Level 2** *Cooperative patients with moderate/complex CHD that are candidate to longitudinal cross-sectional imaging evaluation with CMR and/or CCT → It refers preferably to a specialized center with experience in diagnosis and treatment of CHD.*

This category includes well-established CMR and CCT indications for all simple CHDs in children (e.g., single pulmonary vein anomalies), most moderate complexity (e.g., follow-up in Tetralogy of Fallot or aortic coarctation), and some complex CHDs in both children and adults. They generally have largely accepted protocols but imaging modality choice, post-processing and timing are based on the specific information required for patient management (clinical conditions, need for sedation, and previous imaging) or on local availability and expertise. This category also includes limited cases of CHD with uncertain CMR or CCT indications.

It is recommended to coordinate these decisions with a dedicated team (Cardiologists, Cardiac Surgeons, Cardiovascular Radiologists, Anesthesiologists) committed to long-term collaboration within a referral center [10, 11, 55].

**Level 3** *Uncooperative patients and complex CHD proposed for “optional” and technically difficult cross-sectional imaging investigation with CMR and/or CCT in potentially unsafe scanning condition → it refers to highly specialized and equipped centers with long-standing experience in CHD imaging and treatment.*

This group encompasses all situations where a high level of complexity is expected, be it because they involve very complex CHD pre/post repair/palliation (e.g., Fontan procedure), fragile patients (e.g., newborns or critical patients), technical (e.g., coronary anomalies in small children) or anesthesiologic (e.g., Williams syndrome) difficulties requiring advanced technology, highly specific protocols and experienced staff, no definite agreement on the indication, imaging modality or timing, or other selected cases.

These circumstances necessitate a multidisciplinary evaluation performed during joint meetings of experts that only a highly dedicated referral center can provide [10, 11, 55].

## Recent advances and future perspective

**Fig. 6:**
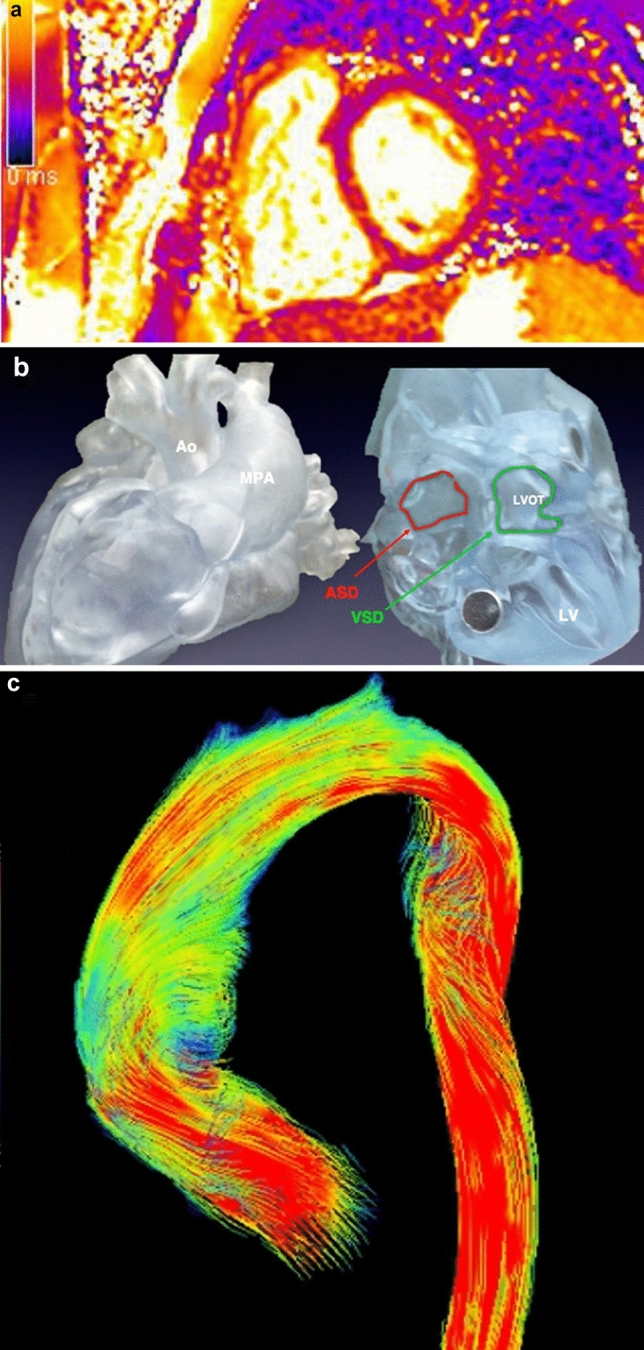
Overview of some of the emergent imaging applications in CHD. T2 map in a short axis basal view of the ventricles in a RV dilatation due to left-to-right shunt (**a**). High complexity CHD 3D printing model: supero-inferior ventricle with a complex relationship of the ventricular septal defect (**b**). 4D flow reconstruction of thoracic aorta: flow streamlines panoramic sagittal oblique visualization in a bicuspid aortic valve patient. Color-coding of different flow velocities (**c**)

In the last decades, the technological developments of CMR and CCT have contributed to their incremental use in CHD. Novel emerging techniques like advanced flow evaluation and reduced acquisition and post-processing times [56] could further expand their role in the near future (Fig. 6).

Four-dimensional flow technology [57–59] allows for time-resolved 3D blood flow direction and velocity assessment in the whole heart and great vessels, rendering flow analysis in any vessel section available in a single acquisition, which is especially appealing in complex CHD. Moreover, advanced parameters such as flow energetics and wall shear stress might be particularly useful in some conditions such as aortopathies.

Tissue mapping parameters (T1,T2, T2* and extracellular volume) offer a quantitative analysis of both focal and overall diffuse myocardial alterations like fibrosis and edema [60–62]. To date their use in CHDs like repaired Tetralogy of Fallot and systemic RV is partially limited by small RV wall thickness, highly exposed to motion artifacts. Future advancements in sequences stability and acquisition speed would probably improve our knowledge of ventricular dysfunction mechanisms and arrhythmogenic risk in these patients [63, 64].

There is growing evidence that myocardial deformation is a more sensitive quantitative assessment of contractile function than ejection fraction. CMR feature tracking [65] measures deformation from simple cine images, overcoming many limitations of myocardial tagging. This opens the possibility to obtain additional diagnostic and prognostic information especially in GUCH, although further research is warranted in this field.

The enhancement of hybrid diagnostic approaches represents a further development in CHD. CMR pulmonary flow regurgitation quantification and invasive pressure measurements [66] are already performed in some centers. CMR guidance of interventional cardiac catheterization has been shown feasible and could be especially useful for electrophysiology procedures, although several issues must still be resolved [67].

A new emerging application in pediatric imaging is fetal CMR [68]. Until recently, this modality was mostly based on static anatomical images. Thanks to advances in fetal cardiac gating techniques, functional imaging is now possible. The combination of flow imaging with oxygen saturation derived from mapping measurements within large fetal vessels allows for calculation of fetal oxygen delivery, consumption, and extraction fraction, providing the only currently available non-invasive insights into fetal hemodynamics [69, 70].

3D printing-prototyping is an ideal manufacturing process for creating patient-specific anatomical models. Its use in CHD is expanding both for surgical and interventional planning and for patients' and families' education [71]. Finally, developments in artificial intelligence and machine/deep learning, including new methods such as radiomics, have a promising role in medical imaging but are still in their early stages [72].

## Summary statement

**Table:** 

	Level 1	Level 2	Level 3
Levels of recommendation of CMR/CCT in CHD			
Definition	*Cooperative Adolescent/adult patients with simple/moderate CHD that require additional cross-sectional imaging investigation*	*Cooperative patients with moderate/complex CHD candidate to longitudinal cross-sectional imaging evaluation*	*Uncooperative patients and complex CHD proposed for “optional” and technically difficult cross-sectional imaging investigation in potentially unsafe scanning condition*
Refer to	*Any center with cardiovascular imaging experience*	*Specialized center with experience in the diagnosis and treatment of CHD*	*Tertiary center with long-standing experience in the diagnosis and treatment of CHD*
